# A case of a superficial circumflex iliac perforator‐intercostal artery perforator (SCIP‐ICAP) compound flap for necrotizing soft tissue infection

**DOI:** 10.1002/ccr3.3320

**Published:** 2020-09-10

**Authors:** Kiichi Furuse, Motoi Kato, Yuya Morishita, Tomoyo Kumagai, Shuichi Nakatsukasa, Tomoyuki Kuwata

**Affiliations:** ^1^ Department of Plastic and Reconstructive Surgery Asahi General Hospital Chiba Japan

**Keywords:** necrotizing soft tissue infection, SCIP‐ICAP compound flap

## Abstract

Even in NSTI patients with many comorbidities, it is possible to save both the life and the limb by thorough debridement and suitable reconstruction. SCIP‐ICAP compound flap can be versatile for a massive defect of an upper extremity. A Case of a Supercharged Compound Flap for Necrotizing Soft Tissue Infection

## Graphical abstract

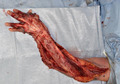

## 1 INTRODUCTION

Superficial circumflex iliac perforator‐intercostal artery perforator (SCIP‐ICAP) compound flap is considered a workhorse flap in the reconstruction of extensive wound cases of the upper extremity for less donor morbidity and thinness. In this report, we present a case of the SCIP‐ICAP compound flap after extensive debridement of the upper extremity with necrotizing soft tissue infection (NSTI). Although the patient had many comorbidities, complete survival of the flap and a satisfactory functional recovery were observed. There has been no previous report of the SCIP‐ICAP compound flap applied for reconstruction of the upper extremity with NSTI. To reconstruct the defect from the dorsum of the hand to the distal upper arm, combination of the flap and the skin graft is a standard treatment; however, the SCIP‐ICAP compound flap is a unique treatment of choice when skin graft is less feasible.

Complex defects of the upper extremity may develop following trauma, tumor resection, radiation injury, and infection.^1^, ^2^ Among infections, necrotizing soft tissue infection (NSTI) requires an extensive debridement,^3^ and various ways of reconstruction can be applied. A superficial circumflex iliac perforator (SCIP) flap is preferred for reconstructing a defect from the dorsum of the hand to the wrist because of its minimal donor site morbidity and flexibility.^4^ However, if the defect extends two‐thirds of the forearm, SCIP flaps may be susceptible to ischemia and distal necrosis.^4^ In such cases, combining skin graft with flap is a popular way of reconstruction. When skin graft is less feasible, extending flaps with supercharge are a treatment of choice.^5^

In this report, we present a case of a supercharged superficial circumflex iliac perforator ‐intercostal artery perforator (SCIP‐ICAP) compound flap after extensive debridement of the upper extremity due to NSTI; it was based on the superficial circumflex iliac artery (SCIA) using ICAP as a supercharge. The defect extended from the dorsum of the hand to the distal upper arm and the flap measured 48 × 7 cm in size. A limited number of SCIP flap cases extended by a supercharged ICAP have been reported in the literature, and there has been no previous report applying the SCIP‐ICAP compound flap for reconstruction of the upper extremity with NSTI. Though the patient was aged and compromised with many comorbidities including angiitis, the flap survived completely and the function of the upper extremity was satisfactory.

## 2 CASE REPORT

**FIGURE 1:**
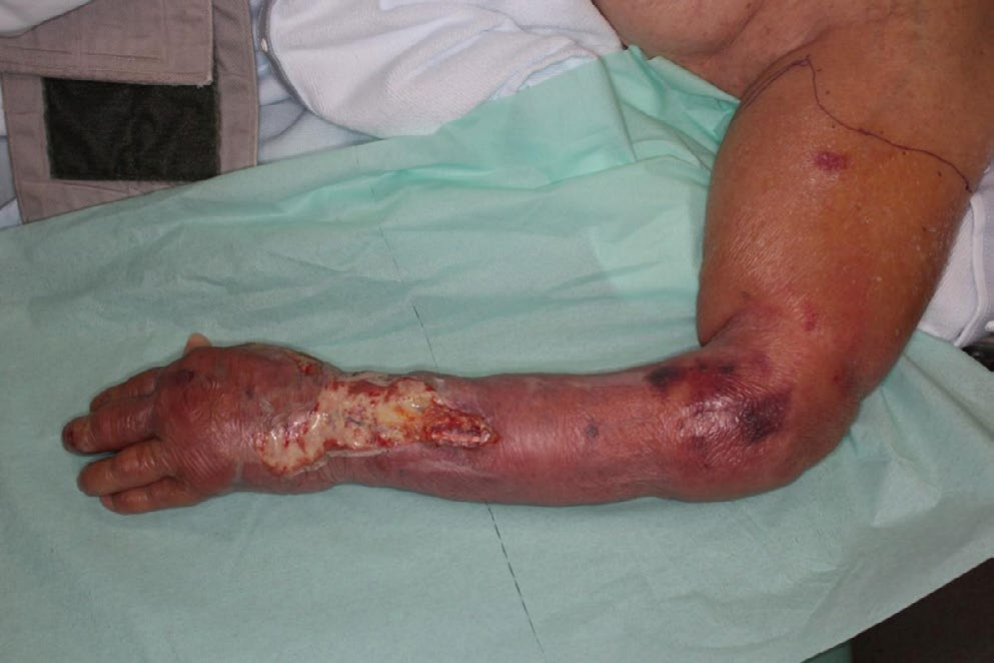
At the time of the first visit, erythema, vesicles, and necrosis from the dorsum of the hand to the distal upper arm was observed

**FIGURE 2:**
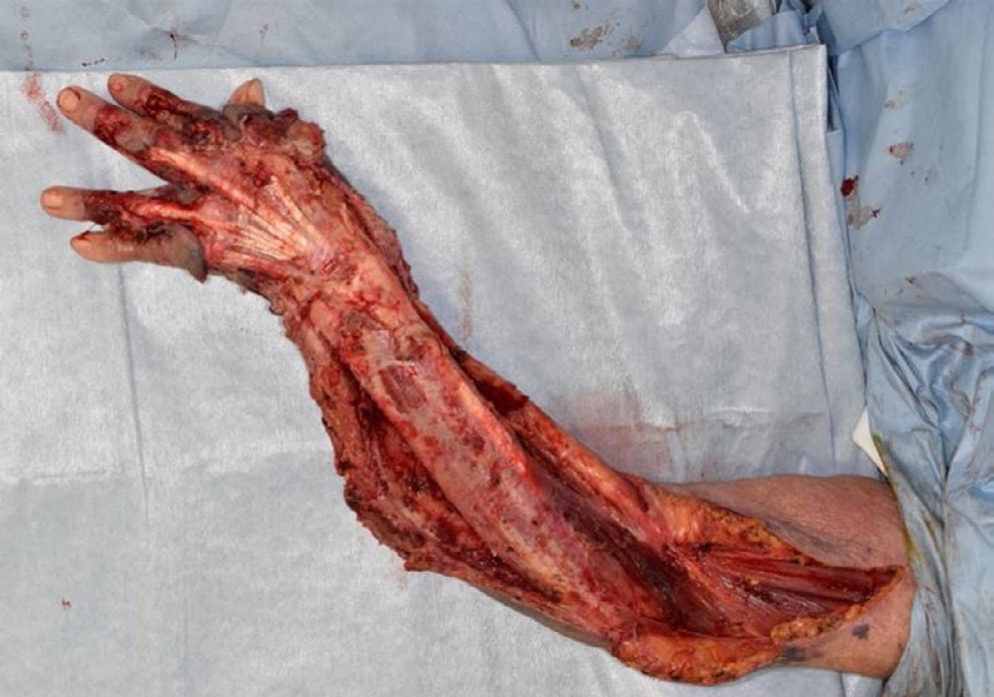
After the debridement, vertical incision was made above the deep fascia

A 78‐year‐old Japanese woman was referred to the emergency department of our hospital with a swollen and painful left arm accompanied by fever. Her underlying diseases included diabetes, rheumatoid arthritis, IgA nephrosis, and microscopic polyangiitis; she was on a prescription of predonin (13 mg/d) and cyclosprine (100 mg/d) as immunosuppressants, and biaspirin (100 mg/d) and persantine (300 mg/d) as anticoagulants. The symptoms started with erythema of the dorsum of the hand 2 days before admission with unknown etiology. She was diagnosed as a suspect of NSTI and referred to our facility. On first visit, erythema and vesicles, and necrosis of the left arm were observed (Figure 1). The affected area was subjected to biopsy, and she was placed on an intravenous infusion of antibiotics. Definitive diagnosis of NSTI was made after 2 bottles of the blood culture and the biopsy revealed Streptococcus pyogenes, and the pathologic analysis yielded fibrinoid necrosis. Though the general status was gradually improved by the conservative treatment, apparent remission was not shown by clinical findings. On the fifth day of admission, extensive debridement was planned. Vertical incision was made from the dorsum of the hand to the upper arm above the deep fascia, the entire fascia was exposed and thoroughly washed with saline (Figure 2). Seventeen days after debridement, signs of improvements were attained by daily wound treatment as shown by local clinical findings and the reconstruction by pedicled SCIP‐ICAP compound flap was performed.

### 2.1 Preoperative assessment and surgical procedure

**FIGURE 3:**
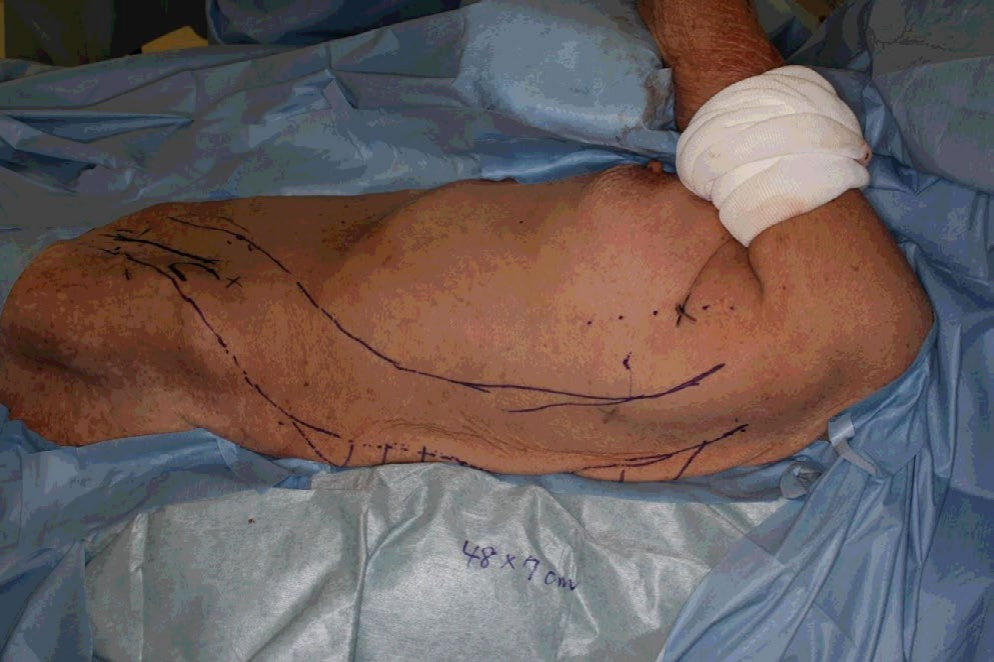
Flap design. SCIP‐ICAP combined flap 48 × 7 cm in size was designed along the left SCIA

**FIGURE 4:**
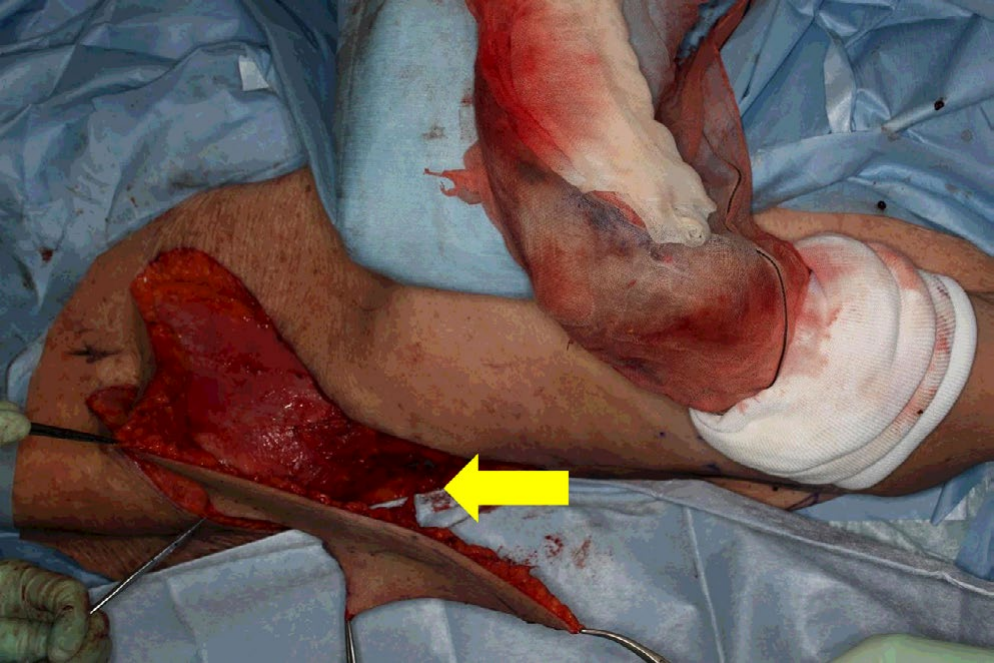
Flap elevation. Yellow arrow: ICAP

In the supine position, a SCIP‐ICAP combined flap was designed along the SCIA from the left groin area toward the posterior axillary line via the anterior superior iliac spine (ASIS) (Figure 3). The flap size was 48 × 7 cm. The flap was elevated from the groin region above the deep fascia of the abdominal external oblique muscle. ICAP was preserved and elevated with the flap (Figure 4). Just before the flap was detached, 2 mL of the fluorescent dye, indocyanine green (ICG, Diagnogreen; Daiichi Pharmaceutical) (0.25 mg/mL) was injected intravenously with the ICAP clamped. As expected, the ICG injection showed the poor vasculature of the distal tip, therefore, both the artery and the vein of the ICAP were anastomosed to ensure the vasculature of the compound flap. A left radial artery perforator and the accompanying vein (checked by ultrasound before the operation) were dissected. The artery and the vein were 1.0 and 2.0 mm, respectively, in diameter (which were almost equal to the ICAP); in addition, they were anastomosed in an end‐to‐end fashion with a 10‐0 nylon suture. The vessel wall was so fragile that the artery had to re‐anastomosed in the course of the operation. The whole flap was checked to ensure it was contrasted by ICG injection and covered the entire area of defect without any tension to the pedicle. The operation time was 5 hours and 27 minutes, and intraoperative bleeding was 210 mL including saline wash.

### 2.2 Postoperative course

**FIGURE 5:**
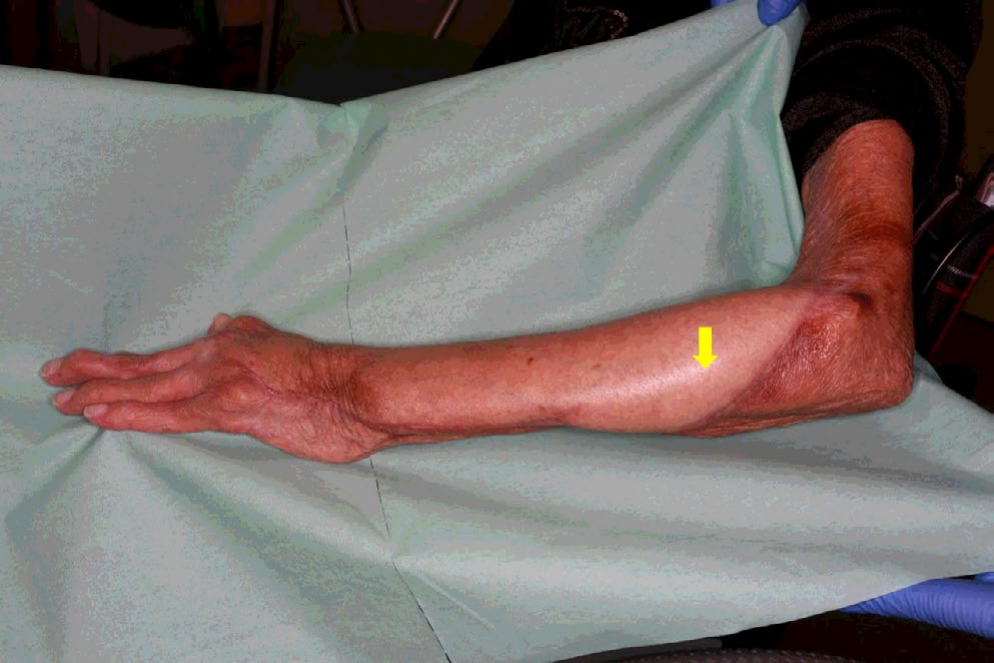
After the pedicle detachment, the flap survived fully, and no infection recurrence was observed. Yellow arrow: the location of the supercharging anastomosis

No anticoagulants nor vasodilators were administered during and after operation. The left upper limb was fixed in place wearing chest binder around the left elbow with cushion in the left axilla and cubital fossa. The pedicle detachment was performed 18 days postoperatively. The flap survived fully (Figure 5), and the patient was discharged from the hospital with no infection recurrence. There was no apparent deformity nor dysfunction 1 year after operation except for rheumatoid deformity.

## 3 DISCUSSION

The concept of the compound flap was defined by Harii et al in 1981 for extensive and complicated wound cases.^5^ Up until now, various cases of compound flaps have been described in the literature and their classification has been introduced by Hallock and Koshima.^6^, ^7^ According to this nomenclature, a supercharged SCIP‐ICAP compound flap was performed. Most reports of the ICAP flap include the upper ICAPs (4‐8th) for reconstruction of the breast and upper arm.^8^, ^9^ Application of the lower ICAPs as reported in this case was described by Iida et al as sensate SCIP flap or ICAP based propeller flap.^10^ A limited number of SCIP flap cases extended by ICAP supercharge have been reported in the literature. However, to the best of our knowledge, there has been no report of the application of a supercharged SCIP‐ICAP flap for a defect in the upper extremity following debridement due to NSTI.

Among various choices of reconstruction, a pedicled compound flap with supercharge was chosen in this case because the defect lied over the wrist and elbow joints; the risk of thrombosis at the site of anastomosis was high, and a difficulty of the patient being in a relaxed condition after the operation was expected. Skin graft was not selected because it was unlikely to survive the tendon exposure and the high risk of infection recurrence and postoperative contracture. A pedicled flap was preferred to a free flap since the recipient vessel could be fragile due to various underlying conditions such as infections, debridement, and many comorbidities. Furthermore, shorter operative time was more desirable due to her poor general condition. Due to the high risk of failure in microsurgical anastomosis as previously reported in a vasculitis case,^11^ we had to re‐anastomose due to microthrombosis during the operation.

The pedicled latissimus dorsi flap could be an alternative; however, the SCIP flap was preferred to cover the dorsum of the hand (which was most susceptible to infection) with the proximal part of the flap. The abdominal flap was another choice of reconstruction as a massive compound flap; however, the caliber discrepancy could be a problem in supercharging with a perforator.

ICAP and perforator in the forearm were considered to match feasibly, because of their similar sizes and decent numbers. The lowest ICAP was cranially located 2‐5 and 7‐11 cm posterior to the ASIS; from that point, ICAPs were cranially identified every 2.5‐3 cm, and they could be included in SCIP flap. The average diameter was reported as 0.7 ± 0.24 mm.^10^, ^12^ On the contrary, the mean number of the radial artery perforators was reported as 10 and 8 on the radial and ulnar side, respectively. The average diameters were 1.11 and 0.86 mm on the distal and proximal side, respectively^13^; these matched the diameter of the ICAPs. Thus, application of SCIP‐ICAP compound flap could be versatile for a massive defect from the dorsum of the hand to the proximal upper arm.

Necrotizing soft tissue infection is a life‐threatening disease characterized by a rapid spreading necrosis. Rapid and thorough debridement are mandatory, and the reconstruction of the vast defect is challenging. In this case, debridement was performed on the fifth day of admission, which was rather later than usual. This was because there was a slow exacerbation of the local clinical findings and safer debridement could be expected when the general status became better due to conservative treatment. Even in a presented case with high risk, working in collaboration with other specialists makes it possible save both the life and the limb by thorough debridement. NSTI is a relatively rare disease and further studies on more cases are therefore necessary to establish the most ideal treatment or method of reconstruction.

## 4 CONCLUSION

A case of a supercharged pedicled SCIP‐ICAP compound flap with satisfactory function recovery (after extensive debridement of the upper extremity with NSTI) was reported. Even in an aged NSTI patient with many comorbidities, it is possible to save both the life and the limb by thorough debridement and reconstruction. Application of SCIP‐ICAP compound flap could therefore be versatile for a massive defect from the dorsum of the hand to the proximal upper arm by supercharging ICAPs with radial artery perforators.

## CONFLICT OF INTEREST

All the authors report no conflicts of interests.

## AUTHOR CONTRIBUTIONS

KF: wrote the initial draft of the manuscript. MK: assisted in the preparation of the manuscript. All other authors contributed to the collection and interpretation of data and the critical review of the manuscript. All authors approved the final version of the manuscript. All authors agree to be accountable for all aspects of the work and will ensure that questions related to the accuracy or integrity of any part of the work are appropriately investigated and resolved.

## ETHICAL APPROVAL

The patient provided informed consent for the publication of this report. The procedures followed were in accord with the Helsinki Declaration of 1975.
